# (20*R*)-24-Bromo-5β-cholane

**DOI:** 10.1107/S1600536809014585

**Published:** 2009-04-25

**Authors:** Kamal Aziz Ketuly, A. Hamid A. Hadi, Seik Weng Ng

**Affiliations:** a Department of Chemistry, University of Malaya, 50603 Kuala Lumpur, Malaysia

## Abstract

In the title compound (5*S*,8*R*,9*R*,10*R*,13*S*,14*S*,17*R*,20*R*)-24-bromo-5β-cholane, C_24_H_41_Br, the fused-chair conformation of the cyclo­hexane *A*/*B* ring junction is *cis* with a 5β-H configuration.

## Related literature

For the isostructural chloro analog, see: Cox *et al.* (2001 ▶).
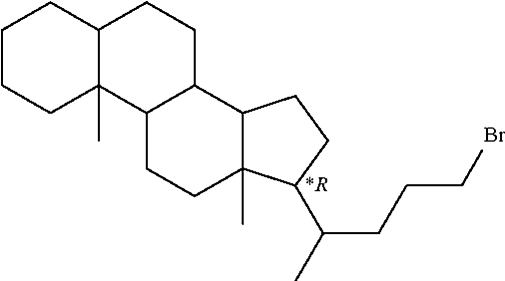


## Experimental

### 

#### Crystal data

C_24_H_41_Br
*M* *_r_* = 409.48
Orthorhombic, 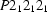

*a* = 7.4797 (2) Å
*b* = 9.9094 (3) Å
*c* = 29.3371 (8) Å
*V* = 2174.5 (1) Å^3^
*Z* = 4
Mo *K*α radiation
μ = 1.90 mm^−1^
*T* = 100 K
0.30 × 0.10 × 0.03 mm

#### Data collection

Bruker SMART APEX diffractometer
Absorption correction: multi-scan (*SADABS*; Sheldrick, 1996 ▶) *T*_min_ = 0.606, *T*_max_ = 0.746 (expected range = 0.767–0.945)
15233 measured reflections
4988 independent reflections
4263 reflections with *I* > 2σ(*I*)
*R*_int_ = 0.048

#### Refinement

*R*[*F* ^2^ > 2σ(*F* ^2^)] = 0.035
*wR*(*F* ^2^) = 0.075
*S* = 0.99
4988 reflections
226 parameters
H-atom parameters constrained
Δρ_max_ = 0.33 e Å^−3^
Δρ_min_ = −0.28 e Å^−3^
Absolute structure: Flack (1983 ▶), 2124 Friedel pairs
Flack parameter: 0.018 (8)

### 

Data collection: *APEX2* (Bruker, 2008 ▶); cell refinement: *SAINT* (Bruker, 2008 ▶); data reduction: *SAINT*; program(s) used to solve structure: *SHELXS97* (Sheldrick, 2008 ▶); program(s) used to refine structure: *SHELXL97* (Sheldrick, 2008 ▶); molecular graphics: *X-SEED* (Barbour, 2001 ▶); software used to prepare material for publication: *publCIF* (Westrip, 2009 ▶).

## Supplementary Material

Crystal structure: contains datablocks global, I. DOI: 10.1107/S1600536809014585/tk2433sup1.cif

Structure factors: contains datablocks I. DOI: 10.1107/S1600536809014585/tk2433Isup2.hkl

Additional supplementary materials:  crystallographic information; 3D view; checkCIF report

## Figures and Tables

**Fig. 1.**
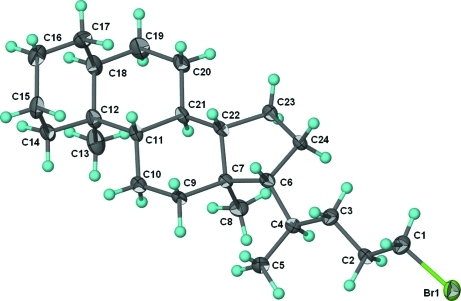
Thermal ellipsoid plot (Barbour, 2001) of C24H41Br at the 70% probability level. Hydrogen atoms are drawn as spheres of arbitrary radius.

## supplementary crystallographic information

### Experimental

The procedure employed was that used for synthesizing 5β-cholan-24-yl chloride, but with phosphorus tribromide in place of phosphorus trichloride. Phosphorus tribromide (0.06 ml, 0.65 mmol) in dichloromethane (10 ml) was added to 24-hydroxy-5β-cholane (22 mg, 0.65 mmol) in dichloromethane (10 ml). The mixture was kept at 283–288 K under stirring for 12 h and then made basic with sodium bicarbonate solution. The organic compound was extracted with hexane–ethyl acetate (4:1 v/v). The solvent was removed to give a yellow oil, which gradually solidified (135 mg). The compound was dissolved in hexane and chromatographed on silica gel (10 g). The first fraction gave the pure compound, m.p. 335–338 K, when the solvent was allowed to evaporate.

### Refinement

Hydrogen atoms were placed at calculated positions (C–H 0.98–1.00 Å) and were treated as riding on their parent carbon atoms, with *U*(H) set to 1.2–1.5 times *U*_eq_(*C*).

### Crystal data

**Table:**

C_24_H_41_Br	*F*(000) = 880
*M**_r_* = 409.48	*D*_x_ = 1.251 Mg m^−^^3^
Orthorhombic, *P*2_1_2_1_2_1_	Mo *K*α radiation, λ = 0.71073 Å
Hall symbol: P 2ac 2ab	Cell parameters from 3210 reflections
*a* = 7.4797 (2) Å	θ = 2.8–24.5°
*b* = 9.9094 (3) Å	µ = 1.90 mm^−^^1^
*c* = 29.3371 (8) Å	*T* = 100 K
*V* = 2174.5 (1) Å^3^	Plate, colorless
*Z* = 4	0.30 × 0.10 × 0.03 mm

### Data collection

**Table:**

Bruker SMART APEX diffractometer	4988 independent reflections
Radiation source: fine-focus sealed tube	4263 reflections with *I* > 2σ(*I*)
graphite	*R*_int_ = 0.048
ω scans	θ_max_ = 27.5°, θ_min_ = 2.2°
Absorption correction: multi-scan (*SADABS*; Sheldrick, 1996)	*h* = −9→9
*T*_min_ = 0.606, *T*_max_ = 0.746	*k* = −12→12
15233 measured reflections	*l* = −37→38

### Refinement

**Table:**

Refinement on *F*^2^	Secondary atom site location: difference Fourier map
Least-squares matrix: full	Hydrogen site location: inferred from neighbouring sites
*R*[*F*^2^ > 2σ(*F*^2^)] = 0.035	H-atom parameters constrained
*wR*(*F*^2^) = 0.075	*w* = 1/[σ^2^(*F*_o_^2^) + (0.0372*P*)^2^] where *P* = (*F*_o_^2^ + 2*F*_c_^2^)/3
*S* = 0.99	(Δ/σ)_max_ = 0.001
4988 reflections	Δρ_max_ = 0.33 e Å^−^^3^
226 parameters	Δρ_min_ = −0.28 e Å^−^^3^
0 restraints	Absolute structure: Flack (1983), 2124 Friedel pairs
Primary atom site location: structure-invariant direct methods	Flack parameter: 0.018 (8)

### Special details

**Table:**

Geometry. All esds (except the esd in the dihedral angle between two l.s. planes) are estimated using the full covariance matrix. The cell esds are taken into account individually in the estimation of esds in distances, angles and torsion angles; correlations between esds in cell parameters are only used when they are defined by crystal symmetry. An approximate (isotropic) treatment of cell esds is used for estimating esds involving l.s. planes.

### Fractional atomic coordinates and isotropic or equivalent isotropic displacement parameters (Å^2^)

**Table:**

	*x*	*y*	*z*	*U*_iso_*/*U*_eq_	
Br1	0.12174 (4)	0.77061 (3)	−0.054189 (9)	0.02429 (8)	
C1	0.0806 (4)	0.6772 (3)	0.00361 (9)	0.0224 (6)	
H1A	0.1165	0.7370	0.0290	0.027*	
H1B	−0.0487	0.6581	0.0069	0.027*	
C2	0.1835 (4)	0.5463 (3)	0.00693 (9)	0.0177 (6)	
H2A	0.3133	0.5658	0.0079	0.021*	
H2B	0.1594	0.4906	−0.0204	0.021*	
C3	0.1301 (4)	0.4678 (2)	0.04965 (8)	0.0193 (5)	
H3A	−0.0002	0.4505	0.0486	0.023*	
H3B	0.1543	0.5246	0.0767	0.023*	
C4	0.2281 (3)	0.3324 (2)	0.05535 (10)	0.0173 (5)	
H4	0.2221	0.2835	0.0256	0.021*	
C5	0.4269 (3)	0.3583 (3)	0.06637 (9)	0.0240 (7)	
H5A	0.4892	0.2718	0.0695	0.036*	
H5B	0.4362	0.4090	0.0950	0.036*	
H5C	0.4814	0.4104	0.0416	0.036*	
C6	0.1334 (3)	0.2460 (2)	0.09118 (8)	0.0151 (5)	
H6	0.1236	0.3020	0.1194	0.018*	
C7	0.2146 (3)	0.1081 (3)	0.10554 (9)	0.0149 (5)	
C8	0.2666 (4)	0.0269 (3)	0.06281 (9)	0.0211 (6)	
H8A	0.3166	−0.0603	0.0720	0.032*	
H8B	0.3559	0.0773	0.0453	0.032*	
H8C	0.1602	0.0121	0.0439	0.032*	
C9	0.3702 (4)	0.1130 (2)	0.13960 (8)	0.0175 (5)	
H9A	0.3418	0.1785	0.1640	0.021*	
H9B	0.4792	0.1449	0.1238	0.021*	
C10	0.4070 (3)	−0.0258 (3)	0.16101 (9)	0.0200 (6)	
H10A	0.4509	−0.0878	0.1370	0.024*	
H10B	0.5029	−0.0163	0.1841	0.024*	
C11	0.2430 (3)	−0.0883 (3)	0.18361 (9)	0.0158 (5)	
H11	0.2062	−0.0241	0.2082	0.019*	
C12	0.2789 (3)	−0.2260 (3)	0.20769 (9)	0.0210 (6)	
C13	0.3411 (4)	−0.3321 (3)	0.17341 (10)	0.0308 (7)	
H13A	0.2498	−0.3435	0.1498	0.046*	
H13B	0.3600	−0.4182	0.1891	0.046*	
H13C	0.4533	−0.3027	0.1593	0.046*	
C14	0.4248 (3)	−0.2139 (3)	0.24460 (9)	0.0228 (6)	
H14A	0.4535	−0.3055	0.2560	0.027*	
H14B	0.5344	−0.1770	0.2304	0.027*	
C15	0.3754 (4)	−0.1259 (3)	0.28485 (9)	0.0246 (6)	
H15A	0.4739	−0.1270	0.3074	0.030*	
H15B	0.3588	−0.0317	0.2744	0.030*	
C16	0.2049 (4)	−0.1755 (3)	0.30718 (10)	0.0300 (7)	
H16A	0.1701	−0.1125	0.3318	0.036*	
H16B	0.2262	−0.2652	0.3210	0.036*	
C17	0.0533 (4)	−0.1856 (3)	0.27254 (10)	0.0284 (7)	
H17A	0.0218	−0.0940	0.2618	0.034*	
H17B	−0.0534	−0.2246	0.2876	0.034*	
C18	0.1038 (3)	−0.2733 (3)	0.23142 (9)	0.0248 (6)	
H18	0.1264	−0.3664	0.2433	0.030*	
C19	−0.0515 (4)	−0.2839 (3)	0.19738 (11)	0.0302 (7)	
H19A	−0.0243	−0.3549	0.1747	0.036*	
H19B	−0.1615	−0.3106	0.2138	0.036*	
C20	−0.0845 (3)	−0.1502 (3)	0.17263 (10)	0.0239 (7)	
H20A	−0.1298	−0.0829	0.1947	0.029*	
H20B	−0.1777	−0.1637	0.1491	0.029*	
C21	0.0841 (3)	−0.0953 (3)	0.15005 (9)	0.0166 (6)	
H21	0.1174	−0.1568	0.1244	0.020*	
C22	0.0536 (3)	0.0461 (3)	0.13073 (9)	0.0156 (5)	
H22	0.0308	0.1058	0.1576	0.019*	
C23	−0.1020 (4)	0.0685 (2)	0.09833 (8)	0.0184 (6)	
H23A	−0.2170	0.0703	0.1150	0.022*	
H23B	−0.1069	−0.0031	0.0748	0.022*	
C24	−0.0613 (3)	0.2077 (3)	0.07660 (9)	0.0174 (6)	
H24A	−0.1472	0.2763	0.0877	0.021*	
H24B	−0.0703	0.2023	0.0430	0.021*	

### Atomic displacement parameters (Å^2^)

**Table:**

	*U*^11^	*U*^22^	*U*^33^	*U*^12^	*U*^13^	*U*^23^
Br1	0.02565 (13)	0.02078 (12)	0.02645 (13)	0.00045 (12)	−0.00377 (13)	0.00517 (11)
C1	0.0273 (18)	0.0216 (13)	0.0184 (14)	0.0042 (12)	0.0029 (11)	0.0008 (11)
C2	0.0125 (13)	0.0209 (13)	0.0198 (14)	−0.0004 (11)	−0.0018 (11)	0.0013 (11)
C3	0.0215 (13)	0.0207 (12)	0.0156 (12)	0.0007 (12)	0.0015 (14)	−0.0002 (10)
C4	0.0148 (13)	0.0203 (12)	0.0169 (12)	0.0011 (10)	0.0006 (12)	0.0010 (13)
C5	0.0177 (15)	0.0284 (15)	0.0258 (16)	−0.0027 (11)	0.0000 (11)	0.0082 (12)
C6	0.0133 (11)	0.0166 (13)	0.0155 (11)	−0.0001 (12)	−0.0003 (10)	−0.0027 (9)
C7	0.0125 (13)	0.0173 (13)	0.0150 (13)	0.0006 (11)	−0.0004 (11)	−0.0007 (10)
C8	0.0208 (15)	0.0229 (14)	0.0196 (16)	0.0036 (11)	0.0036 (12)	−0.0038 (11)
C9	0.0101 (12)	0.0223 (13)	0.0201 (13)	−0.0011 (13)	−0.0003 (13)	0.0032 (10)
C10	0.0151 (15)	0.0228 (13)	0.0223 (14)	−0.0009 (11)	−0.0023 (12)	0.0019 (11)
C11	0.0144 (13)	0.0151 (13)	0.0180 (13)	0.0021 (10)	−0.0041 (11)	−0.0013 (11)
C12	0.0184 (13)	0.0169 (12)	0.0278 (14)	0.0013 (12)	−0.0053 (11)	0.0030 (13)
C13	0.0326 (19)	0.0209 (14)	0.0389 (18)	0.0089 (13)	−0.0092 (15)	0.0002 (13)
C14	0.0150 (13)	0.0214 (14)	0.0321 (15)	0.0004 (11)	−0.0032 (11)	0.0099 (13)
C15	0.0285 (15)	0.0217 (13)	0.0236 (14)	−0.0023 (15)	−0.0098 (15)	0.0064 (11)
C16	0.0326 (17)	0.0320 (16)	0.0254 (16)	−0.0009 (14)	−0.0012 (14)	0.0102 (13)
C17	0.0218 (15)	0.0290 (16)	0.0343 (17)	−0.0024 (12)	0.0024 (13)	0.0150 (13)
C18	0.0205 (13)	0.0162 (11)	0.0378 (15)	−0.0044 (14)	−0.0080 (12)	0.0103 (12)
C19	0.0254 (15)	0.0214 (14)	0.0439 (18)	−0.0079 (14)	−0.0070 (14)	0.0090 (15)
C20	0.0151 (15)	0.0234 (14)	0.0331 (16)	−0.0040 (11)	−0.0063 (12)	0.0059 (12)
C21	0.0129 (14)	0.0166 (12)	0.0203 (13)	0.0005 (10)	−0.0044 (11)	−0.0001 (10)
C22	0.0113 (12)	0.0155 (13)	0.0202 (14)	0.0018 (10)	−0.0022 (11)	−0.0046 (11)
C23	0.0147 (14)	0.0220 (13)	0.0184 (13)	−0.0029 (12)	−0.0040 (12)	0.0009 (10)
C24	0.0116 (12)	0.0237 (14)	0.0168 (13)	0.0018 (10)	−0.0005 (10)	0.0018 (11)

### Geometric parameters (Å, °)

**Table:**

Br1—C1	1.956 (3)	C12—C14	1.542 (3)
C1—C2	1.511 (4)	C12—C18	1.555 (4)
C1—H1A	0.9900	C13—H13A	0.9800
C1—H1B	0.9900	C13—H13B	0.9800
C2—C3	1.528 (3)	C13—H13C	0.9800
C2—H2A	0.9900	C14—C15	1.514 (4)
C2—H2B	0.9900	C14—H14A	0.9900
C3—C4	1.538 (3)	C14—H14B	0.9900
C3—H3A	0.9900	C15—C16	1.515 (4)
C3—H3B	0.9900	C15—H15A	0.9900
C4—C6	1.530 (3)	C15—H15B	0.9900
C4—C5	1.542 (3)	C16—C17	1.526 (4)
C4—H4	1.0000	C16—H16A	0.9900
C5—H5A	0.9800	C16—H16B	0.9900
C5—H5B	0.9800	C17—C18	1.534 (4)
C5—H5C	0.9800	C17—H17A	0.9900
C6—C7	1.553 (3)	C17—H17B	0.9900
C6—C24	1.565 (3)	C18—C19	1.536 (4)
C6—H6	1.0000	C18—H18	1.0000
C7—C9	1.535 (4)	C19—C20	1.530 (4)
C7—C8	1.539 (3)	C19—H19A	0.9900
C7—C22	1.541 (4)	C19—H19B	0.9900
C8—H8A	0.9800	C20—C21	1.525 (3)
C8—H8B	0.9800	C20—H20A	0.9900
C8—H8C	0.9800	C20—H20B	0.9900
C9—C10	1.537 (3)	C21—C22	1.529 (4)
C9—H9A	0.9900	C21—H21	1.0000
C9—H9B	0.9900	C22—C23	1.519 (4)
C10—C11	1.526 (4)	C22—H22	1.0000
C10—H10A	0.9900	C23—C24	1.550 (3)
C10—H10B	0.9900	C23—H23A	0.9900
C11—C21	1.545 (3)	C23—H23B	0.9900
C11—C12	1.560 (4)	C24—H24A	0.9900
C11—H11	1.0000	C24—H24B	0.9900
C12—C13	1.527 (4)		
			
C2—C1—Br1	112.46 (18)	C12—C13—H13A	109.5
C2—C1—H1A	109.1	C12—C13—H13B	109.5
Br1—C1—H1A	109.1	H13A—C13—H13B	109.5
C2—C1—H1B	109.1	C12—C13—H13C	109.5
Br1—C1—H1B	109.1	H13A—C13—H13C	109.5
H1A—C1—H1B	107.8	H13B—C13—H13C	109.5
C1—C2—C3	110.9 (2)	C15—C14—C12	114.8 (2)
C1—C2—H2A	109.5	C15—C14—H14A	108.6
C3—C2—H2A	109.5	C12—C14—H14A	108.6
C1—C2—H2B	109.5	C15—C14—H14B	108.6
C3—C2—H2B	109.5	C12—C14—H14B	108.6
H2A—C2—H2B	108.1	H14A—C14—H14B	107.5
C2—C3—C4	114.1 (2)	C16—C15—C14	110.8 (2)
C2—C3—H3A	108.7	C16—C15—H15A	109.5
C4—C3—H3A	108.7	C14—C15—H15A	109.5
C2—C3—H3B	108.7	C16—C15—H15B	109.5
C4—C3—H3B	108.7	C14—C15—H15B	109.5
H3A—C3—H3B	107.6	H15A—C15—H15B	108.1
C6—C4—C3	110.0 (2)	C15—C16—C17	111.0 (2)
C6—C4—C5	113.3 (2)	C15—C16—H16A	109.4
C3—C4—C5	109.7 (2)	C17—C16—H16A	109.4
C6—C4—H4	107.9	C15—C16—H16B	109.4
C3—C4—H4	107.9	C17—C16—H16B	109.4
C5—C4—H4	107.9	H16A—C16—H16B	108.0
C4—C5—H5A	109.5	C16—C17—C18	112.2 (2)
C4—C5—H5B	109.5	C16—C17—H17A	109.2
H5A—C5—H5B	109.5	C18—C17—H17A	109.2
C4—C5—H5C	109.5	C16—C17—H17B	109.2
H5A—C5—H5C	109.5	C18—C17—H17B	109.2
H5B—C5—H5C	109.5	H17A—C17—H17B	107.9
C4—C6—C7	119.9 (2)	C19—C18—C17	111.3 (2)
C4—C6—C24	112.27 (19)	C19—C18—C12	111.5 (2)
C7—C6—C24	103.01 (19)	C17—C18—C12	112.9 (2)
C4—C6—H6	107.0	C19—C18—H18	106.9
C7—C6—H6	107.0	C17—C18—H18	106.9
C24—C6—H6	107.0	C12—C18—H18	106.9
C9—C7—C8	110.8 (2)	C20—C19—C18	111.8 (2)
C9—C7—C22	107.0 (2)	C20—C19—H19A	109.3
C8—C7—C22	112.3 (2)	C18—C19—H19A	109.3
C9—C7—C6	116.4 (2)	C20—C19—H19B	109.3
C8—C7—C6	109.7 (2)	C18—C19—H19B	109.3
C22—C7—C6	100.12 (19)	H19A—C19—H19B	107.9
C7—C8—H8A	109.5	C19—C20—C21	112.4 (2)
C7—C8—H8B	109.5	C19—C20—H20A	109.1
H8A—C8—H8B	109.5	C21—C20—H20A	109.1
C7—C8—H8C	109.5	C19—C20—H20B	109.1
H8A—C8—H8C	109.5	C21—C20—H20B	109.1
H8B—C8—H8C	109.5	H20A—C20—H20B	107.8
C7—C9—C10	111.9 (2)	C22—C21—C20	111.4 (2)
C7—C9—H9A	109.2	C22—C21—C11	108.0 (2)
C10—C9—H9A	109.2	C20—C21—C11	112.1 (2)
C7—C9—H9B	109.2	C22—C21—H21	108.4
C10—C9—H9B	109.2	C20—C21—H21	108.4
H9A—C9—H9B	107.9	C11—C21—H21	108.4
C11—C10—C9	113.4 (2)	C23—C22—C21	118.7 (2)
C11—C10—H10A	108.9	C23—C22—C7	103.9 (2)
C9—C10—H10A	108.9	C21—C22—C7	115.3 (2)
C11—C10—H10B	108.9	C23—C22—H22	106.0
C9—C10—H10B	108.9	C21—C22—H22	106.0
H10A—C10—H10B	107.7	C7—C22—H22	106.0
C10—C11—C21	111.1 (2)	C22—C23—C24	103.7 (2)
C10—C11—C12	114.4 (2)	C22—C23—H23A	111.0
C21—C11—C12	112.4 (2)	C24—C23—H23A	111.0
C10—C11—H11	106.1	C22—C23—H23B	111.0
C21—C11—H11	106.1	C24—C23—H23B	111.0
C12—C11—H11	106.1	H23A—C23—H23B	109.0
C13—C12—C14	107.5 (2)	C23—C24—C6	106.6 (2)
C13—C12—C18	110.1 (2)	C23—C24—H24A	110.4
C14—C12—C18	107.8 (2)	C6—C24—H24A	110.4
C13—C12—C11	110.9 (2)	C23—C24—H24B	110.4
C14—C12—C11	111.8 (2)	C6—C24—H24B	110.4
C18—C12—C11	108.8 (2)	H24A—C24—H24B	108.6
			
Br1—C1—C2—C3	−172.81 (18)	C16—C17—C18—C19	179.2 (2)
C1—C2—C3—C4	179.6 (2)	C16—C17—C18—C12	−54.6 (3)
C2—C3—C4—C6	−165.3 (2)	C13—C12—C18—C19	−64.9 (3)
C2—C3—C4—C5	69.4 (3)	C14—C12—C18—C19	178.1 (2)
C3—C4—C6—C7	−175.8 (2)	C11—C12—C18—C19	56.8 (3)
C5—C4—C6—C7	−52.6 (3)	C13—C12—C18—C17	168.9 (2)
C3—C4—C6—C24	63.2 (3)	C14—C12—C18—C17	52.0 (3)
C5—C4—C6—C24	−173.6 (2)	C11—C12—C18—C17	−69.4 (3)
C4—C6—C7—C9	79.4 (3)	C17—C18—C19—C20	70.3 (3)
C24—C6—C7—C9	−155.1 (2)	C12—C18—C19—C20	−56.7 (3)
C4—C6—C7—C8	−47.5 (3)	C18—C19—C20—C21	53.6 (3)
C24—C6—C7—C8	78.0 (2)	C19—C20—C21—C22	−172.9 (2)
C4—C6—C7—C22	−165.7 (2)	C19—C20—C21—C11	−51.7 (3)
C24—C6—C7—C22	−40.2 (2)	C10—C11—C21—C22	−53.7 (3)
C8—C7—C9—C10	−68.8 (3)	C12—C11—C21—C22	176.6 (2)
C22—C7—C9—C10	53.9 (3)	C10—C11—C21—C20	−176.8 (2)
C6—C7—C9—C10	164.8 (2)	C12—C11—C21—C20	53.5 (3)
C7—C9—C10—C11	−55.0 (3)	C20—C21—C22—C23	−53.6 (3)
C9—C10—C11—C21	54.2 (3)	C11—C21—C22—C23	−177.1 (2)
C9—C10—C11—C12	−177.2 (2)	C20—C21—C22—C7	−177.7 (2)
C10—C11—C12—C13	−62.0 (3)	C11—C21—C22—C7	58.8 (3)
C21—C11—C12—C13	65.9 (3)	C9—C7—C22—C23	169.8 (2)
C10—C11—C12—C14	57.9 (3)	C8—C7—C22—C23	−68.4 (3)
C21—C11—C12—C14	−174.1 (2)	C6—C7—C22—C23	47.9 (2)
C10—C11—C12—C18	176.8 (2)	C9—C7—C22—C21	−58.6 (3)
C21—C11—C12—C18	−55.3 (3)	C8—C7—C22—C21	63.2 (3)
C13—C12—C14—C15	−172.6 (2)	C6—C7—C22—C21	179.6 (2)
C18—C12—C14—C15	−53.9 (3)	C21—C22—C23—C24	−165.6 (2)
C11—C12—C14—C15	65.5 (3)	C7—C22—C23—C24	−36.0 (2)
C12—C14—C15—C16	56.6 (3)	C22—C23—C24—C6	10.0 (3)
C14—C15—C16—C17	−54.7 (3)	C4—C6—C24—C23	149.5 (2)
C15—C16—C17—C18	54.7 (3)	C7—C6—C24—C23	19.2 (2)
